# Survival from testicular cancer in England and Wales up to 2001

**DOI:** 10.1038/sj.bjc.6604598

**Published:** 2008-09-23

**Authors:** R A Huddart

**Affiliations:** 1 The Institute of Cancer Research, 15 Cotswold Road, Belmont, Sutton, Surrey SM2 5NG, UK

For many years testicular cancer has been the prime example of the tumour that is chemocurable, even when metastatic. The disappointment in oncology is that these results have so far not been replicated in the more common solid tumours. Why this should be is not clear but germ-cell tumours retain sensitivity to chemotherapy *in vitro* and a number of mechanisms including reduced DNA repair capacity and proneness to apoptosis have been proposed (Mayer *et al*, 2003).

Most patients with testicular cancer present after finding a lump in the testicle that may or may not be painful. A small proportion of patients present with symptoms of metastatic disease. With the exception of some patients with metastatic disease, initial treatment after first assessment is to remove the tumour by inguinal orchidectomy. Patients are staged by tumour marker estimation and CT scan. In most patients (60–70%) no further evidence of disease is found. These patients are still at risk of relapse and are managed by careful observation or by administration of low toxicity adjuvant therapy. For patients with metastatic disease the mainstay of treatment is combination chemotherapy. Though some patients were cured as early as the 1960s with chemotherapy (Table 1) it was the advent of cisplatin in the late 1970s and then etoposide in the early 1980s, which transformed the prognosis of the disease. Together with bleomycin, these two drugs make up the gold standard chemotherapy schedule for testicular cancer, BEP. This schedule, first developed in the early 1980s, rapidly superseded previous treatments and has not been improved on since in any randomised studies.

Perhaps it is a surprise, then, that there has been continued improvement in survival since the 1980s through to the late 1990s with commensurate improvement in older patients and in the socially deprived. Why should this be when the chemotherapy (BEP) used is essentially the same as in the early 1980s?

One explanation is likely to be earlier presentation of patients, resulting from improved education and awareness of testicular cancer in both patients and their doctors. Since the early 1990s several charities such as The Everyman Campaign and Cancer Research UK have been vocal in raising the awareness and profile of testicular cancer, with evidence that the general population are both more aware of testicular cancer and practice testicular self-examination.

This has been coupled with recent targets to see new patients within 2 weeks and operate within a further 4 weeks. In a review of our testicular database at the Royal Marsden the median time from symptoms to orchidectomy has fallen for Stage I non-seminomatous germ-cell tumours (NSGCT) and seminoma from 79 and 70.5 days, respectively, in 1970–1974 to 54 and 41 days in 1995–1999 (*P*<0.05 comparing 1995–1999 with the rest; Alan Horwich, unpublished observations; Figure 1). Similar results have been reported from Yorkshire when the average time of symptoms to GP vists reduced from 5 to 2 weeks (Vasudev *et al*, 2004).

In a rapidly growing tumour like TGCT it would be anticipated that reductions in presentation delays would lead to reduced tumour size and earlier stage at diagnosis, findings recently reported both in the United Kingdom (Bhardwa *et al*, 2005) and Europe (Sonneveld *et al*, 1999; Lackner *et al*, 2006). In the UK study from St Bartholomew's Hospital in London the average tumour size at presentation has fallen from 4 cm in 1984–1995 to 2.5 cm between 1999 and 2002 (*P*=0.002) whereas over the same time periods, the proportion of patients with Stage I disease increased from 57 to 77% (Figure 1). This is important as Stage I patients have anticipated survival rates in excess of 99% (Schmoll *et al*, 2004) and within Stage I, seminoma size is an important prognostic factor for subsequent relapse (Warde *et al*, 2002). In addition, and anecdotally, several major UK centres have reported fewer patients with most advanced prognostic metastatic disease (Bhala *et al*, 2004).

Stage migration may be one explanation for improved survival but even in those presenting with metastatic disease there has been some suggestion of improved outcomes. Mead *et al* (1997) reported a pooled analysis of patients with NSGCT and seminoma who were largely treated in the 1980s. This study defined prognostic categories. For NSGCT the good prognosis group had an expected 5-year survival of 91%, intermediate prognosis of 79% and poor prognosis of 48%. These have become the internationally accepted benchmark survival figures. Several reports have suggested improved outcomes over time (Sonneveld *et al*, 2001; Bhala *et al*, 2004) and (Muramaki *et al*, 2004) with the greatest improvements being seen in the poorer prognostic groups. For instance, in the report from Bhala *et al* from Sheffield the 5-year survival between 1989 and 2001 for good, intermediate and poor prognostic patients was 95, 82 and 57% respectively. A number of reports using more intensive chemotherapy have reported even better results in poor prognosis patients with survival rates exceeding 75% (Bower *et al*, 1997; Christian *et al*, 2003).

Why should survival improve in these patient groups? Some groups as noted above, have used more intensive treatment but these approaches are yet to show improved survival over standard BEP treatment in randomised trials. Over this time period there has been increased awareness of the importance of dose intensity, reduction in the use of carboplatin, which was common in the 1980s but shown to be less active in randomised trials (Horwich *et al*, 1997) and improved supportive care, for example, antibiotic care and use of growth factors. This has been associated with a move to treat testicular cancer patients in specialist centres. This is based on data that patients treated in larger specialist centres fair better than patients treated elsewhere (Harding *et al*, 1993; Collette *et al*, 1999). The report of Collette *et al*, who showed, within the context of a EORTC/MRC randomised trial that centres recruiting five or more patients had better survival than centres recruiting fewer patients (2-year survival 77 *vs* 62%; HR=1.85; *P*=0.01), is particularly important. A number of possible explanations were highlighted in this paper: patients from the larger recruiting centre having trends for a higher dose intensity, fewer treatment delays, lower toxic death rate (6 *vs* 13%) and more surgical resections (65 *vs* 52%). This report and an earlier study from the west of Scotland (Harding *et al*, 1993) with similar results have driven the movement to increased centralization of both surgical and non-surgical management. It is now recommended in the United Kingdom that testicular cancer services are delivered by networks with not less than 2 million population.

Where do we go from here? There is some scope to improve outcomes for the most socially deprived sections of the community, to achieve the results equivalent to the wealthiest sections of our population. We do also have to start thinking of the longer term: Not what the outcome is over 5 or 10 years but over a lifetime in this young group of patients. Recent reports of increased late cardiac morbidity and second malignancy (Huddart, 2003; Zagars *et al*, 2004; Travis *et al*, 2005) in long-term survivors are a concern. This means we have to start to look at our current strategies to minimise these risks. We do not want to win this battle and lose the war.

## Summary

The improved outcomes for UK testicular cancer patients should be welcomed. This is likely to be because of earlier diagnosis, standardisation of treatment, treatment in specialist centres and improved supportive care. Further study should focus on ensuring that the best standards of care are available to the entire population and to minimise the very long-term risks of treatment.

## Figures and Tables

**Figure 1:**
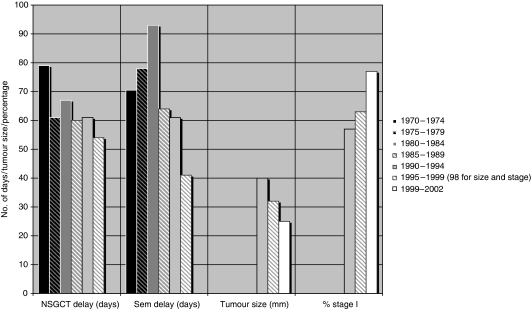
Changes in delays in diagnosis, tumour size and stage at presentation over time. Based on unpublished observations from the Royal Marsden testicular tumour database and data on tumour size and stage at presentation data from St Bartholomew's Hospital, London (Bhardwa *et al*, 2005).

**Table 1:** Progress in chemotherapy schedules for testicular cancer; outcomes from chemotherapy in metastatic disease

**Estimations**	**1970–1974**	**1975–1979**	**1980–1984**	**1985–1989**	**1990–1994**	**1995–1999 (98 for size and stage)**	**1999–2002**
NSGCT delay (days)	79	61	67	60	61	54	—
Sem delay (days)	70.5	78	93	64	61	41	—
Tumour size (mm)	—	—	—	—	40	32	25
% stage I	—	—	—	—	57	63	77

NSCGT=non-seminomatous germ-cell tumours.
